# Multisystem Inflammatory Syndrome in Adults (MIS-A) Reported in Patient with Hematological Malignancy: A Case Report

**DOI:** 10.3390/jpm13020178

**Published:** 2023-01-19

**Authors:** Elżbieta Kalicińska, Paula Jabłonowska, Tomasz Wróbel

**Affiliations:** Department of Hematology, Blood Neoplasms and Bone Marrow Transplantation, Wrocław Medical University, 50-367 Wrocław, Poland

**Keywords:** multisystem inflammatory syndrome in adults, hematological malignancy, case report, immunosuppression

## Abstract

Background: The coronavirus disease 2019 (COVID-19) has persisted for over 2 years worldwide and has long-term effects on the health and quality of life of convalescents. Multisystem inflammatory syndrome, primary observed in children is currently increasingly recognized in adults. Immunopathology might play a crucial role in the pathogenesis of multisystem inflammatory syndrome in adults (MIS-A); therefore, the occurrence of MIS-A in non-immunocompetent patients is a significant challenge in diagnosis and treatment. Case presentation: We described a 65-year-old patient with Waldenström’s macroglobulinemia (WM) who suffered from MIS-A after COVID-19 and was successfully treated with high doses of immunoglobulins and steroids. Conclusion: Our study presents for the first time a case of MIS-A in a hematological patient with a broad spectrum of symptoms reflecting multiorgan damage and suggests the long-term consequences of MIS-A as persistent immune dysregulation involving T-cell response.

## 1. Background

The COVID-19 pandemic has had a huge impact on public health worldwide. The clinical COVID-19 manifestations resulting from dysregulation of renin–angiotensin–aldosterone system (RAAS), endothelial dysfunction and impaired immune response [1]. The long-term consequences of COVID-19 are still poorly understood. The diagnosis of multisystem inflammatory syndrome in adults is difficult due to the convergent clinical presentation with biphasic acute COVID-19 or long COVID-19 syndrome [1]. Moreover, patients may present a different spectrum of symptoms, which makes the diagnosis even more challenging.

In immunosuppressed patients, reduced baseline blood counts and a delayed immune system response may further complicate the interpretation of the observed laboratory abnormalities and organ dysfunctions. However, due to immunosuppression, the diagnosis of MIS-A and the timely implementation of treatment have become a priority in effectively reducing hyperinflammation and reversing adverse organ changes.

## 2. Case Presentation

The focus of this report is a 65-year-old man with Waldenström’s macroglobulinemia, treated with obinutuzumab from October 2020 to April 2021, with partial response achievement, subsequently undergoing maintenance treatment with obinutuzumab (the monoclonal antibody was given every 2 months), with the last dose given on 15 December 2021. Waldenström’s macroglobulinemia was diagnosed in November 2007. In previous treatment, he received 2 lines of immunochemotherapy (in 2007 and in 2008).

He was admitted to our hospital, on 24 February 2022, due to conjunctivitis and severe thrombocytopenia. Two days before admission, he had a fever of 38.5 °C. He had received two doses of COVID-19 vaccine, one in March and one in June 2021. He had a history of SARS-CoV-2 infection on 7th of January with a mild course. The patient was ambulatory and treated with molnupiravir. After antiviral treatment, he received a negative SARS-CoV-2 PCR result. Moreover, he had a history of arterial hypertension and prostate hypertrophy with no significant family history. On admission, a SARS-CoV-2 PCR test via nasopharyngeal swab was negative. On physical examination, numerous petechiae were present on the skin and mucous membranes. Additionally, features of conjunctivitis were observed. The patient had fever of 38 °C. Laboratory findings on admission revealed severe thrombocytopenia, lymphopenia, elevated inflammatory and coagulation biomarkers (CRP, fibrinogen, D-dimer), and high levels of lactate dehydrogenase (Table 1).

**Figure 1:**
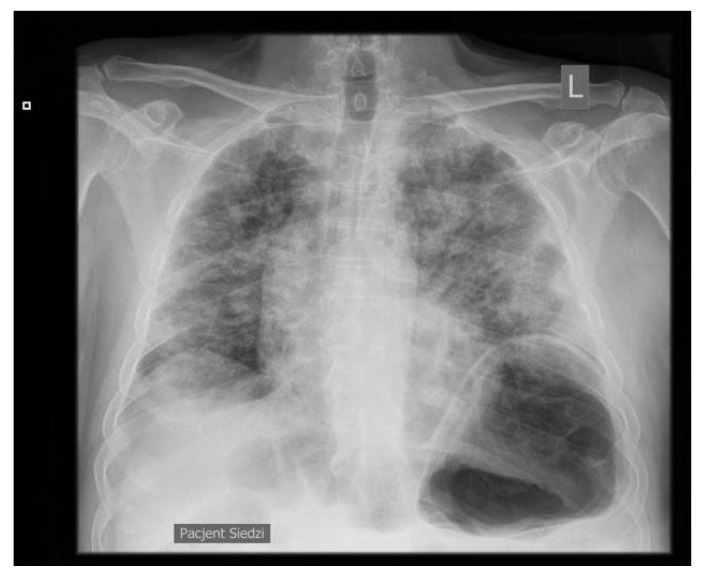
Chest X-ray. L—left side.

On day 3, the patient developed dyspnea, with bilateral rales and crackles over the lungs. On the chest X-ray, numerous signs of bilateral heterogenous shading with unclear etiology were observed (Figure 1).

**Figure 2:**
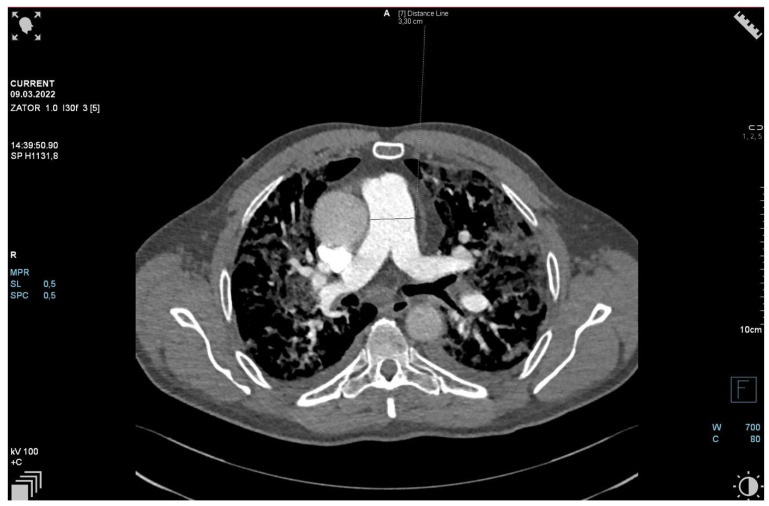
Angio-CT scan.

**Figure 3:**
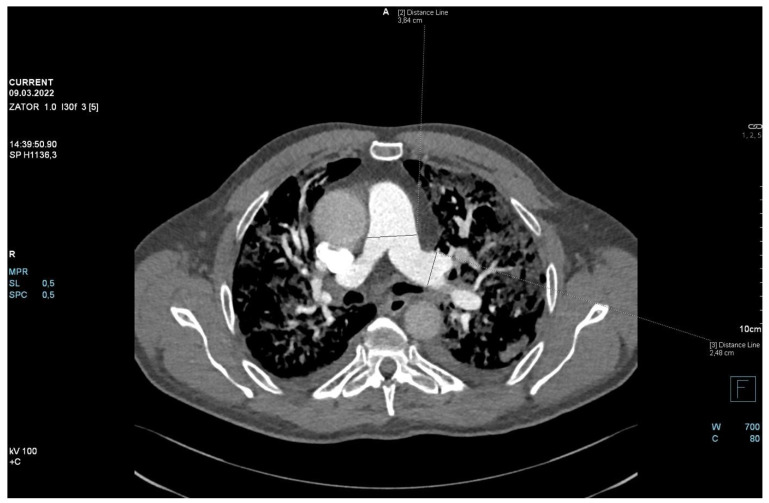
Angio-CT scan.

**Figure 4:**
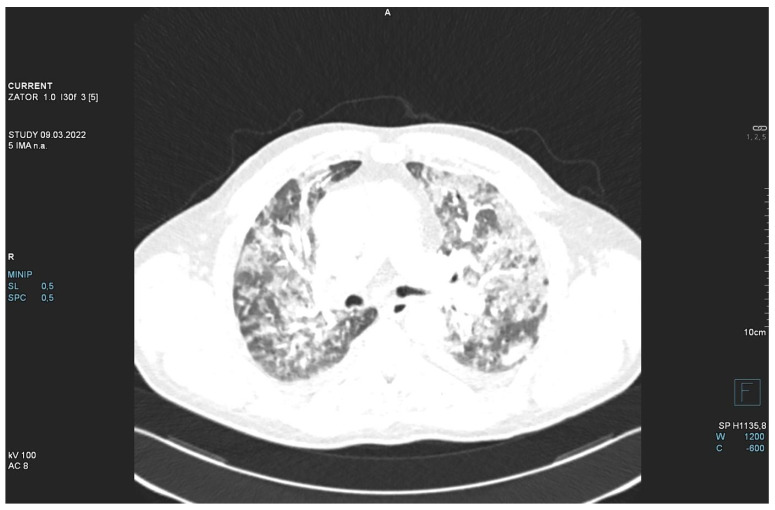
Angio-CT scan.

The patient required oxygen therapy, and initially, due to a lack of diagnosis, he received empirically broad-spectrum antibiotics (meropenem, linezolide). On laboratory testing, a further increase in inflammatory and coagulation biomarkers (CRP, ferritin, interleukin-6, D-Dimer, fibrinogen) was observed. Blood cultures as well as a respiratory PCR multitest were negative. Due to the dynamic increase in D-dimers and the elevation of cardiac biomarkers (BNP, NT-proBNP) along with dyspnea, an angio-CT was performed. It excluded pulmonary embolism, but it revealed widening of the pulmonary trunk and pulmonary arteries, as well as alveolar compaction in both lungs and bilateral pleural effusion. Pulmonary edema was diagnosed (Figure 2, Figure 3 and Figure 4).

The patient was treated with diuretics and showed clinical improvement. However, on day 5, he developed neurological complications such as confusion and dizziness. Despite platelets transfusions, the levels of platelets remained extremely low. All presented symptoms (cardiac failure presented as pulmonary edema, severe thrombocytopenia refractory to transfusions, conjunctivitis, high inflammatory and coagulation markers, and neurological symptoms), in a patient who 5 weeks prior had undergone COVID-19 treatment, strongly indicated MIS-A. The treatment with high doses of intravenous immunoglobulin (40 g per day) and high doses of methylprednisolone (250 mg per day) were initiated and were continued during the next 5 days. On day 10, inflammatory markers and natriuretic peptides decreased. Laboratory results showed a significant increase in platelet count (Table 1). During the next 5 days, steroids were reduced, the control chest X-ray showed no evidence of pulmonary edema.

Three months later, patient returned to his daily activities. Levels of inflammatory markers, natriuretic peptides, and levels of platelets returned to normal values. No signs of neurological impairment were observed. Despite the normalization of lymphocyte counts and their subsets 3 months after MIS-A, the CD4/CD8 ratio assessed by peripheral blood immunophenotyping remained in a disturbed proportion (Table 2).

## 3. Discussion and Conclusions

We demonstrated, for the first time, post-COVID-19 MIS-A in a patient with hematological malignancy successfully treated with high doses of immunoglobulins and steroids.

Our patient presented with hyperinflammatory multiorgan dysfunction that occurred approximately 3–4 weeks after SARS-CoV-2 infection, after viral clearance confirmed by a negative PCR swab on admission.

Systematic reviews which described patients with MIS-A highlighted that most of them presented with elevated coagulation and inflammatory biomarkers, fever, hypotension, cardiac dysfunction, dyspnea, and diarrhea [1]. The wide spectrum of symptoms makes it difficult to recognize MIS in adults. The diagnosis of MIS in immunosuppressed adult patients appears to be a particular challenge.

According to CDC definition, MIS-A is diagnosed in patients >21 years, hospitalized >24 h, with a positive SARS-CoV-2 result in the last 2–12 weeks, who meet the following criteria: 1) subjective or documented fever (>38.0 °C), at least one primary (severe cardiac illness, rash and non-purulent conjunctivitis) and at least two secondary criteria (new onset neurologic signs and symptoms, shock or hypotension, abdominal pain or vomiting or diarrhea, thrombocytopenia) [2]. Additionally, laboratory criteria should be present (elevated at least two inflammatory biomarkers—C-reactive protein, procalcitonin, ferritin, IL-6, erythrocyte sedimentation rate). Alternative diagnoses should be excluded [2].

Our patient met the criteria of MIS-A diagnosis according to the CDC definition [2]. The patient presented new symptoms of hyperinflammation and multiorgan dysfunction one month after mild COVID-19 with a period of full recovery. Two days before admission, he had a fever of 38.5 °C. The respiratory insufficiency observed on day 3 was caused by congestion and pulmonary edema due to cardiac failure with a sudden increase in natriuretic peptides levels. Therefore, it could not be confused with two-phase acute SARS-CoV-2 infection. During hospitalization, there was a significant decrease in the number of platelets and lymphocytes. These parameters returned to baseline values after treatment. However, transfusion-resistant thrombocytopenia, especially in hematological patients with MIS-A, seems to be of particular importance in the context of endothelial damage [3]. This phenomenon, combined with excessive inflammation along with deep lymphopenia, can cause pulmonary edema leading to respiratory symptoms. On the other hand, endothelial dysfunction could occur in other organs and result in their failure. Our patient had symptoms of cardiac decompensation with no alternative causes of cardiovascular deterioration.

The pathophysiology of multisystem inflammatory syndrome after COVID-19 is still unclear, especially in adults in whom MIS-A is rarely observed. The potential mechanism includes delayed, inappropriate immune reaction which induces inflammation [1,4]. The observed hyperinflammation in adults may be triggered on the one hand by immunosenescence, and on the other hand by immunosuppression as a consequence of underlying diseases [1,5]. Current data from a systematic review described 221 adult patients with MIS-A [1]. Most of these patients were young, without comorbidities. In patients with underlying diseases, especially those with a weakened immune system, the diagnosis of MIS-A becomes a challenge.

Our patient was immunosuppressive due to treatment with monoclonal antibody (obinutuzumab) and his underlying disease (WM), which affects mainly B lymphocytes. Long-term treatment with obinutuzumab results in a reduction in CD4 T cells and NK cells [6].

Therefore, patients treated with CD20 monoclonal antibody have both dysregulated innate and humoral immunity resulted in impaired production of neutralizing antibodies. This may lead to latent infection with low viral load present in multiple organs [7]. On the other hand, MIS-A is characterized by a dysregulation of the immune response [8]. This may explain the complicated and severe course of MIS-A in our patient and may suggest a deep, potentialized immunological defect which is pronounced in hematological patients with post-COVID-19 inflammatory syndrome.

COVID-19 results in T-cell lymphopenia [9]. In patients with hematological malignancies, the major immune abnormalities relate in particular to the depletion of CD4 T-cell counts [10]. The dysfunction of the immunological system in our patient, expressed as a low CD4/CD8 ratio, persisted for 3 months after recovery and indicated severe marked reconstitution in the course of post-COVID-19 inflammatory syndrome. Previous studies indicate the possibility of the persistence of T-lymphopenia in the group of patients with excessive inflammation during COVID-19 up to 12 months after acute infection [11]. It can therefore be speculated that the hyperinflammatory state during MIS-A further exacerbates the persistence of T-cell perturbation, especially in immunosuppressive patients.

Further investigations of immunopathology after MIS-A will help us to understand the long-term consequences of COVID-19 in hematological patients and clarify whether immune disturbances are persistent or transient, and how long they persist, especially in patients with pre-existing immunosuppression due to hematological malignancies and applied treatment.

## Figures and Tables

**Table 1:** Longitudinal laboratory characteristic of WM patient with MIS-A.

Variable	Normal Range	2 Months before MIS-A	Day 1	Day 3	Day 5	Day 7	Day 10	after 3 Months
C-reactive protein [mg/L]	0.2–5.0	1.6	20.6	117	172	104	47	7.1
Procalcitonin [ng/mL]	0–0.05	--	0.09	0.3	0.66	0.59	0.3	0.05
Interleukin-6 [pg/mL]	0–5.9	--	36	197	282	11	2	1.2
Ferritin [ug/L]	20–290	111	390	1670	1810	1520	858	101
Fibrinogen [g/L]	2–4.39	2.77	5.16	5.89	6.22	4.39	3.44	3.77
D-dimers [ug/mL]	0–0.5	0.242	0.667	1.63	5.02	15.29	6.7	0.97
LDH [U/L]	125–220	169	295	560	588	642	440	206
White blood cells [10^3^/uL]	4–10	3.83	2.36	6.81	8.16	12.58	9.17	5.99
Lymphocytes [10^3^/uL]	1.5–3.5	1.44	0.59	0.65	0.59	0.59	0.71	1.76
Neutrophiles [10^3^/uL]	2.5–6	1.7	1.31	5.58	7.19	11.67	8.14	3.36
Hemoglobin [g/dL]	14–18	14	13.7	13.7	11.6	11.4	10.3	13.2
Platelets [10^3^/uL]	140–440	63	3	4	2	15	43	75
BNP [pg/mL]	0–125	--	102	166	426	194	111	25.4
NT-proBNP [pg/mL]	0–125	--	231	659	2354	899	305	68.6

**Table 2:** Lymphocyte profile in WM patient after MIS-A recovery.

T CD3+ Lymphocytes			
CD3+: 100%	CD8+: 65%	CD5+: 91%	CD56 + CD16+: negative
CD7+: 85%	CD4+: 35%	CD4+/CD8+ ratio: 0.5	

Legend: WBC: 6420/μL; Lymphocytes: 1541/μL (normal range: 1000–2800); NK cells: 308/μL (normal range: 90–600); CD3+: 1233/μL (normal range: 700–2100); CD4+: 431/μL (normal range: 300–1400); CD8+: 814/μL (normal range: 200–900).

## Data Availability

The data and materials are available from the corresponding author.

## Abbreviations

B-type natriuretic peptide (BNP), Centers for Disease Control and Prevention (CDC), computed tomography angiogram (angio-CT), coronavirus disease 2019 (COVID-19), C-reactive protein (CRP), interleukin 6 (IL-6), multisystem inflammatory syndrome in adults (MIS-A), natural killer cells (NK cells), N-terminal prohormone of B-type natriuretic peptide (NT-proBNP), renin–angiotensin–aldosterone system (RAAS), polymerase chain reaction (PCR), Waldenström’s macroglobulinemia (WM).
